# Composition and physiological functions of the porcine colostrum

**DOI:** 10.1111/asj.13618

**Published:** 2021-08-19

**Authors:** Ryo Inoue, Takamitsu Tsukahara

**Affiliations:** ^1^ Laboratory of Animal Science, Department of Applied Biological Sciences, Faculty of Agriculture Setsunan University Hirakata Japan; ^2^ Kyoto Institute of Nutrition & Pathology Kyoto Japan

**Keywords:** colostrum, passive immunity, pig, whey protein

## Abstract

The first secretion, 24‐h post parturition of the mammary glands of sows, known as colostrum, is high in protein and low in lactose and fat. As a consequence of an insufficient ingestion of colostrum, more than 50% of piglets fail to reach weaning and die. The composition and some functions of colostrum have been previously reported. For example, colostrum carbohydrates consist of mainly lactose. Lipids in the colostrum are mostly triacylglycerols, but <1% is fatty acids, which may act as homeostasis regulators. Similarly, proteins are found mostly as casein and whey, the latter being ≥80% immunoglobulins. Colostrum‐derived immunoglobulins and bioactive proteins such as azurocidin help the immune system of the piglet fend off infections. In addition, leukocytes and exosomes are other minor but nonetheless equally crucial bioactive components in the porcine colostrum. Modern pig farming has achieved increases in pig productivity and litter size, but this has been accomplished in detriment of the health and the survival rate of piglets. Therefore, porcine colostrum is now even more important in pig farming. In the present review, we discuss the current knowledge on the composition and physiological functions of the porcine colostrum and briefly propose future research directions.

## 1 INTRODUCTION

The colostrum of sows, as that of most mammals, is defined as the first secretion of the mammary gland (Quesnel et al., 2012), usually released during the first 24 h post parturition (Hurley, 2015). Unlike the sow's milk, its colostrum is characterized by a higher concentration of protein and lower concentrations of lactose and fat (Quesnel et al., 2012).

The ingestion of colostrum during the first 24 h is known to be one of the most crucial factors for the healthy development of piglets. For example, it has been reported that the mortality of piglets that ingested less than 100 g of colostrum during the first 24 h is over 60%, whereas that of piglets ingesting more than 200 g is 10% or lower (Quesnel et al., 2012). In addition, Devillers et al. (2011) observed long‐lasting effects of colostrum ingestion on the growth of piglets. These workers also found that the postweaning body weights of piglets ingesting less than 290 g of colostrum in the first 24 h were about 15% lower than average.

The colostrum provides piglets with the energy needed for thermoregulation and body growth (Herpin et al., 2005; Le Dividich et al., 2005), as well as essential growth factors that stimulate the development of organs (Burrin et al., 1992; Xu et al., 2002). Furthermore, due to the unique epitheliochorial structure of the porcine placenta, which prevents the transfer of factors that confer maternal immunity, the colostrum is almost the sole source of passive immunity for piglets (Rooke & Bland, 2002). Thus, deficient ingestion of colostrum likely leads to a high vulnerability of piglets to microbial infections.

Previous studies (Burrin et al., 1992; Ogawa, Tsukahara, et al., 2016) demonstrated that milk secreted 48 h post parturition or formula milk without bioactive components could not replace colostrum from a physiological standpoint. This evidence clearly indicates the uniqueness of the composition and functions of colostrum. Intensive farming of dairy cows worldwide has permitted an easier and more plentifully collection of bovine colostrum. The access to more bovine colostrum has in turn permitted to gather knowledge of its composition not only in a more consistent and but also a faster manner. By contrast, although over the past 20 years analysis of the composition and physiological functions of the porcine colostrum has been steady, the pace of its investigation has been far from ideal. In this review, we aimed to summarize the data available on the composition and physiological functions of the porcine colostrum based on previous works, including studies conducted at these premises.

## 2 COMPOSITION OF THE PORCINE COLOSTRUM

The porcine colostrum consists mainly of proteins, carbohydrates, lipids, and, in lesser degree, minerals, vitamins, leukocytes, and somatic cells (Xu et al., 2002; Zhang et al., 2018). Other studies reported the presence of additional bioactive components such as exosomes (Chen et al., 2014) and bacteria (Martin et al., 2009) in the sow's colostrum. Because the enterocytes in the small intestine of newborn piglets can take up macromolecules via nonspecific pinocytosis, some of the colostral components are likely to be absorbed intact and transferred into the blood circulation in piglets (Clarke & Hardy, 1971; Payne & Marsh, 1962; Westrom et al., 1984). The proportions of colostrum components can differ depending on a variety of factors including pig breed, parity order, and diets, but the trends are generally similar (Luise et al., 2018; Segura et al., 2020; Trevisi et al., 2020). For example, proteins account for approximately 16% of total first‐secreted colostrum (Hurley, 2015; Kemp et al., 2018). Lactose is the main carbohydrate and a major osmole in the sow's colostrum, accounting for about 3% (Hurley, 2015; Kemp et al., 2018). Oligosaccharides can be also found in the porcine colostrum as bioactive components (Tao et al., 2010), with more than 90 of them being identified thus far (Wei et al., 2018). Lipids are the major source of energy in the porcine colostrum, with an average content of about 5% to 8% (Hurley, 2015; Kemp et al., 2018). More than 90% of lipids in the sow's colostrum are triacylglycerols, followed by diacylglycerols (2% to 4%) (Luise et al., 2018). Although free fatty acids are found in less than 1%, it is believed they play an important role in body homeostasis (Waidyatillake et al., 2017). Of these, palmitic, oleic, and linoleic acids are the three major free fatty acids in the porcine colostrum (Luise et al., 2018).

## 3 PROTEINS IN THE PORCINE COLOSTRUM

**FIGURE 1:**
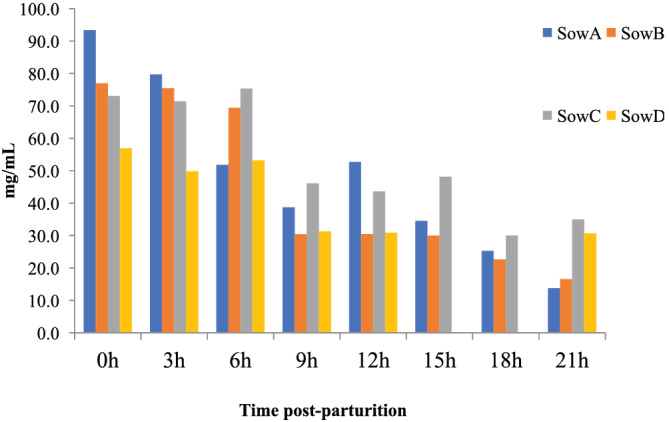
Concentration of IgG in colostrum collected 21 h post parturition. The colostrum was collected from four sows (Landrace × Large White) 0 to 21 h post parturition. IgG concentration was measured by ELISA (unpublished)

About 10% to 20% of the proteins found in the porcine colostrum is casein and the remaining being whey protein (Csapó et al., 1996). In whey protein, immunoglobulin is the most abundant compound (≥80%) (Xu et al., 2002). IgG is the predominant immunoglobulins in the sow's colostrum, followed by IgA (Klobasa et al., 1987; Xu et al., 2002). They are transferred to the piglet's bloodstream via the aforementioned pinocytic activity of small intestinal enterocytes during the first 24–48 h of life (Devillers et al., 2011; Payne & Marsh, 1962). The colostrum‐derived immunoglobulins play a pivotal role in protecting against bacterial and viral infections. For example, previous work demonstrated that maternally derived IgG specific for rotavirus played a significant role in mitigating clinical symptoms following an experimental rotaviral infection of neonatal piglets (Ward et al., 1996). It should be noted that the concentrations of immunoglobulins in the sow's colostrum start to decrease even during 24 h after parturition (Figure 1, Inoue et al., unpublished data), with the decrease in IgG more drastic than that of IgA. For instance, from 0 to 24 h post parturition, the concentration of IgG decreases by 80% to 90% and continues to decrease until around 7 days post parturition. Conversely, the concentration of IgA moderately decreases by 50% to 70% 24 h post parturition, but the decrease almost ceases until 48 h post parturition. Therefore, the concentration of IgA observed in the colostrum 48 h after delivery will likely remain unchanged 42 days post parturition (Klobasa et al., 1987).

Hence, the milk secreted by the sow 7 days post parturition is less abundant in IgG than in IgA (Klobasa et al., 1987). Intriguingly, the concentrations of immunoglobulins in the colostrum can differ even from teat to teat of the same sow. Indeed, a previous study carried out at our facilities demonstrated that the volume of colostrum secretion in the posterior teats was significantly higher than that in the anterior teats and that both IgG and IgA concentrations showed a significant positive correlation with the volume of secretion in the teats (Ogawa, Tsukahara, Tsuruta, et al., 2014). These findings were in agreement with a past report that showed that piglets suckling from the posterior teats had higher daily body weight gains than those suckling from the anterior teats (Kim et al., 2000).

Apart from immunoglobulins, there are many other proteins that are unique to or highly concentrated in the sow's colostrum. Table 1 shows the proteins we previously detected in the sow's colostrum and milk using shotgun proteomic analysis (Ogawa, Tsukahara, Nishibayashi, et al., 2014). In fact, 70 proteins including azurocidin (also known as cationic antimicrobial protein 37 kDa or heparin‐binding protein) were successfully identified as unique or highly concentrated proteins. Azurocidin is produced by neutrophil granules and exerts direct antimicrobial activity (Watorek, 2003). In addition, azurocidin also acts as chemoattractant on immune cells. Separately, it was reported that lactoferrin had a higher concentration in the colostrum than in the milk of sows (Elliot et al., 1984). The existence of antimicrobial agents such as azurocidin and lactoferrin may suggest that porcine colostrum possesses direct and nonspecific antimicrobial activity. It is worth noting that lysozyme, an antimicrobial agent found in the human colostrum, does not seem to be present in the porcine colostrum and milk (Xu et al., 2002).

**TABLE 1:** Unique o highly concentrated proteins in the porcine colostrum

Swiss‐Prot accession number	Protein name	emPAI^a^	Score^b^	emPAI	Score
		Colostrum		Mature milk	
Proteins identified in all samples but highly concentrated in colostrum					
P29700	**Alpha‐2‐HS‐glycoprotein (fragment)**	0.32	202	0.1	22
P18648	**Apolipoprotein A‐I**	0.6	158	0.27	125
O18831	**Growth/differentiation factor 8**	1.28	477	0.28	137
P50828	**Hemopexin**	0.32	204	0.15	77
P01846	**Ig lambda chain C region**	7.41	453	3.58	305
Q8MJ39	**Matrix Gla protein**	2.05	170	0.75	111
P09571	**Serotransferrin**	4.22	1,503	1.39	922
Proteins found only in the colostrum					
Q29197	40S ribosomal protein S9 (fragment)		20		
P62831	60S ribosomal protein L23		21		
A1XQU5	60S ribosomal protein L27		21		
P19205	Acylamino acid‐releasing enzyme		19		
P50578	Alcohol dehydrogenase [NADP(+)]		21		
P50447	**Alpha‐1‐antitrypsin**	0.16	75		
Q8MJ76	**Alpha‐fetoprotein**	0.05	27		
P18650	**Apolipoprotein E**	0.48	160		
P16469	Arachidonate 12‐lipoxygenase, 12S‐type		21		
P00503	Aspartate aminotransferase, cytoplasmic		21		
P80015	**Azurocidin**	0.15	35		
Q95266	Calcium/calmodulin‐dependent protein kinase type II subunit delta		20		
P35750	Calpain‐1 catalytic subunit		23		
Q08092	Calponin‐1		21		
P36887	cAMP‐dependent protein kinase catalytic subunit alpha		19		
A5GFW5	Cas scaffolding protein family member 4		40		
Q28944	**Cathepsin L1**	0.1	41		
P02540	Desmin		26		
Q8MJ30	Dihydropteridine reductase		20		
Q28943	Dihydropyrimidine dehydrogenase [NADP(+)]	0.03	20		
Q6UAQ8	Electron transfer flavoprotein subunit beta	0.14	43		
O97939	**Enamelin**		26		
Q29042	**Ficolin‐1**		26		
A4GVD1	Gap junction gamma‐1 protein		23		
P20305	**Gelsolin (fragment)**		19		
A5A779	Geranylgeranyl transferase type‐2 subunit alpha		31		
P34930	Heat shock 70 kDa protein 1A		29		
A5A8V7	Heat shock 70 kDa protein 1‐like		24		
P12682	**High mobility group protein B1**		19		
Q9GLP0	Integrin beta‐1		23		
P79263	**Inter‐alpha‐trypsin inhibitor heavy chain H4**		42		
O19073	**Interleukin‐18**		23		
P33198	Isocitrate dehydrogenase [NADP], mitochondrial (fragment)		45		
P79287	**Matrix metalloproteinase‐20**		38		
Q865F1	Microsomal triglyceride transfer protein large subunit		29		
P26042	Moesin		31		
Q9TV63	Myosin‐2		44		
Q8MJ49	Osteoclast‐stimulating factor 1		20		
Q7SIB7	Phosphoglycerate kinase 1		21		
O02696	Phosphoinositide 3‐kinase regulatory subunit 5		20		
Q3ZD69	Prelamin‐A/C		23		
Q01580	**Proheparin‐binding EGF‐like growth factor**		35		
P23687	Prolyl endopeptidase		19		
Q2EN75	Protein S100‐A6	0.4	33		
Q7YS91	Protein TBRG4		25		
P26044	Radixin		42		
Q0GFF6	Retinoic acid receptor RXR‐gamma		19		
Q06AT9	RNA‐binding protein 4B		19		
Q3YLA6	Serine/arginine‐rich splicing factor 1		28		
P61292	Serine/threonine‐protein phosphatase PP1‐beta catalytic subunit		22		
D2WKD8	Sodium/potassium‐transporting ATPase subunit alpha‐2	0.03	37		
B8Y466	SRSF protein kinase 3		21		
O97676	Sterol regulatory element‐binding protein 1		27		
A5GFT6	Teashirt homolog 2		33		
P15203	**Transforming growth factor beta‐3**		20		
P50390	**Transthyretin**	0.54	114		
P42639	Tropomyosin alpha‐1 chain		22		
A1Y2K1	Tyrosine‐protein kinase Fyn	0.06	39		
O19064	Tyrosine‐protein kinase JAK2		32		
Q29561	UMP‐CMP kinase		21		
Q867B5	V(D)J recombination‐activating protein 1		27		
P26234	Vinculin		24		
Q8HXL3	WD repeat‐containing protein 62		24		

*Note*: This protein list is a modified version of that of Ogawa, Tsukahara, Nishibayashi, et al. (2014). Proteins listed in bold are secretory proteins according to the Gene Ontology‐cellular component terms.

^a^ The exponentially modified protein abundance index (emPAI) is 10^ (the number of experimentally observed peptides/the calculated number of observable peptides for each protein)‐1.

^b^ A score is −10 × log(*P*), where *P* is the probability that the observed match is a random event. Proteins with twofold or higher emPAI scores in the colostrum than in mature milk were regarded as highly concentrated proteins. Only proteins with *P* values lower than 0.05 are shown. Proteins with no emPAI scores were found in relatively low amounts.

**FIGURE 2:**
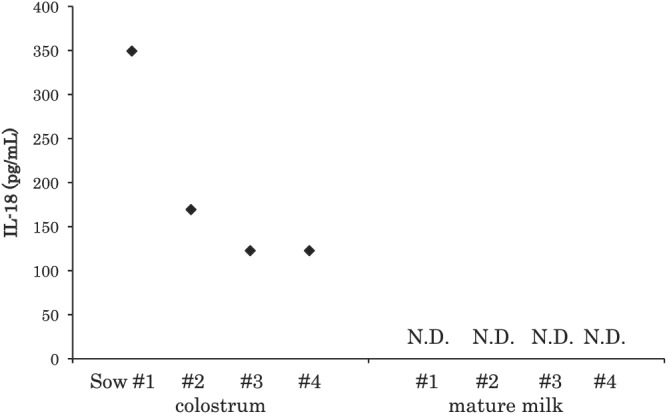
Interleukin‐18 concentration in colostrum and mature milk. Modified from Ogawa, Tsukahara, Nishibayashi, et al. (2014). N.D. indicates the concentration was below detection (39.1 pg/ml)

Interleukin‐18 (IL‐18; Table 1) is a cytokine known to enhance the IL‐12‐driven helper 1 T cell immune response (Muneta et al., 2000). We previously confirmed the presence of IL‐18 in the colostrum but not in the milk of sows (Figure 2; Ogawa, Tsukahara, Nishibayashi, et al., 2014). Nguyen et al. (2007) evaluated the presence of other cytokines, namely, IL‐4, IL‐6, IL‐10, and IL‐12, and found that most of them were higher concentrated in the colostrum than in the milk of sows and possibly transferred to piglets via colostrum. These colostrum‐derived cytokines may also play important roles in the development and maintenance of piglets' immunity.

Proteins related to the epidermal growth factor and the transforming growth factor beta are listed in Table 1. Porcine colostrum has been found to contain higher concentrations of various growth factors than milk (Xu et al., 2002), including the insulin‐like growth factors I and II (Donovan et al., 1994). In general, the concentrations of these growth factors are 5‐ to 10‐fold or even greater in the colostrum than in the milk of sows (Jaeger et al., 1987). These growth factors in the colostrum likely contribute to the development and/or maturation of organs in piglets. However, most work analyzing the growth factors in the porcine colostrum was conducted more than 20 years ago. Therefore, there is a growing consensus on the need to analyze the growth factors in the porcine colostrum using newer, more sensitive analytical methods.

Although a previous study at these premises identified 113 proteins (Ogawa, Tsukahara, Nishibayashi, et al., 2014), a still ongoing study using a more sensitive proteomic analytical technique has detected, so far, the presence of more than 600 proteins in the sow's colostrum (Inoue et al., unpublished data). We expect to find more proteins in the sow's colostrum whose bioactivity is likely yet to be identified.

## 4 LEUKOCYTES IN THE PORCINE COLOSTRUM

The porcine colostrum contains a considerable number of leukocytes. For example, Evans et al. (1982) found an average of 10^7^ leukocyte cells per milliliter of porcine colostrum. The cell types reported by two different studies (Evans et al., 1982; Wuryastuti et al., 1993) were similar (Table 2). Neutrophils and lymphocytes seem to be the predominant cell types in the porcine colostrum. The subproportion of lymphocytes in the colostrum differs from that in peripheral blood of sows. For example, in the porcine colostrum, the proportion of cytotoxic (CD8 positive) and DP (CD4/CD8 double positive) T cells are significantly higher than those in peripheral blood (Hlavova et al., 2014). In contrast, the proportion of helper (CD4 positive) T cells in the porcine colostrum is lower than that in peripheral blood. Based on a flow‐cytometric analysis, Hlavova et al. (2014) concluded that the majority of colostral T cells were central or effector memory cells. Interestingly, based on the gene expression profiles of colostral T cells, previous work at these premises led us to reach a similar conclusion (Ogawa, Okutani, et al., 2016). Therefore, based on this evidence, it is reasonable to theorize that at least some colostral T cells are in fact effector memory cells. This memory phenotype of colostral T cells is useful as the primary protection of piglets against microbial infections, as it has been demonstrated that colostral T cells can be transferred to the bloodstream of piglets (Williams, 1993). An illustrative case is the study by Bandrick et al. (2008), who reported that *Mycoplasma hyopneumoniae*‐specific T cells, transferred to piglets via colostrum from sows vaccinated against this bacterium, participated in the neonatal immune response upon stimulation.

**TABLE 2:** Cell counts in the colostrum of sows

	Evans et al. (1982)	Wuryastuti et al. (1993)
Neutrophils	71.7	64
Macrophages	1.3	5.6
Lymphocytes	26.4	26.5
Eosinophils	0.2	0.7
Epithelial cells	0.4	1.4

*Note*: Data are expressed as percentages of the total cell yields. Large White sows were used in Evans et al. (1982); Yorkshire pure breed, Duroc × Yorkshire crossbreds, and Landrace × Yorkshire crossbreds were used in Wuryastuti et al. (1993).

## 5 EXOSOMES IN THE PORCINE COLOSTRUM

Other bioactive components in the porcine colostrum worth scrutiny are the exosomes. The exosomes are nanosized, endosome‐derived membrane vesicles that are involved in various types of cellular communication (van Niel et al., 2018). The exosomes contain various functional molecules such as mRNA, microRNA, DNA, proteins, and lipids. Although differences in the amounts of exosomes in the porcine colostrum and milk are unclear to date, the exosome quality is likely to be different because the miRNA composition in exosomes changes as lactation carries on (Gu et al., 2012). Similarly, although the functions of colostral exosomes are still mostly unknown, as with other colostral components, they could be transferred to piglets (Alsaweed et al., 2015). Chen et al. (2016) suggested that exosomes in the porcine milk (isolated from milk collected 1–5 days post parturition) stimulated the proliferation of small intestinal epithelial cells in vitro. Moreover, Chen et al. (2016) demonstrated in a mouse model that porcine milk exosomes stimulated the structural development of the small intestine. Recently, Zeng et al. (2021) reported yet another important function of the porcine milk exosomes. In point of fact, these workers demonstrated that small extracellular vesicles, containing exosomes and isolated from milk collected 3–5 days post parturition, promoted intestinal immunoglobulin production by stimulating the expression of polymeric immunoglobulin receptors. This evidence seems to imply that milk exosomes play crucial roles in the growth and/or development of piglets. Nonetheless, as the evidence regarding the functions of porcine milk exosomes is still limited, further investigation is most likely warranted, in particular, that focusing on colostral exosomes, because most available data are on milk exosomes.

## 6 STIMULATION OF THE GROWTH OF VISCERAL ORGANS BY THE PORCINE COLOSTRUM

Unlike mature or formula milk, the colostrum possesses unique physiological functions. For example, the immunoglobulins found in colostrum are well‐known anti‐infectious components that are the primary defense against pathogens of piglets, whose immune systems are not yet fully developed (Quesnel et al., 2012; Xu et al., 2002). The stimulation of the growth of visceral organs could also well be a function unique to the colostrum, because milk alone does not seem to exert such stimulation in piglets (Burrin et al., 1992). The organ whose growth is mostly stimulated by colostrum is the small intestine. For example, according to Widdowson and Crabb (1976), the weight of the small intestine increased about 1.5‐fold during the first 24 h when piglets suckled colostrum, whereas it almost did not increase in piglets given water only. Wang and Xu (1996) compared the weights of the small intestines of 3‐day‐old piglets given either colostrum or lactose. Wang and Xu observed results similar to those of Widdowson and Crabb (1976). In addition, both Widdowson and Crabb (1976) and Wang and Xu (1996) reported that the small intestines were longer in piglets given colostrum than those of piglets having water or lactose only. It must be mentioned that colostral proteins in the process of being absorbed may have accounted for some of the weight of the small intestines of piglets (Widdowson & Crabb, 1976). Nonetheless, in a separate study, Burrin et al. (1992) demonstrated that the small intestines of piglets suckling colostrum showed a higher protein synthesis, when compared with those of piglets given water or milk. This evidence may indicate that colostrum indeed exerts a growth‐stimulating effect on the small intestine.

Data regarding the growth stimulative effect of the colostrum on visceral organs other than the small intestine are still limited. According to Wang and Xu (1996), the colostrum also exerts a growth‐stimulating effect on the large intestine, although to a lesser extent, when compared with that on the small intestine. Burrin et al. (1992) reported that protein contents in liver, pancreas, kidney, and spleen were not significantly different between piglets suckling colostrum or milk, when measured 6 h post delivery. However, they found higher fractional protein and absolute protein synthesis rates in liver and spleen in piglets suckling colostrum than in those suckling milk only. Thus, it seems that while there is no short‐term effect of colostrum on the liver and the spleen, it is possible that the colostrum does stimulate the growth of other organs midterm and long term.

## 7 STIMULATION OF THE DEVELOPMENT OF THE IMMUNE SYSTEM BY PORCINE COLOSTRUM

**FIGURE 3:**
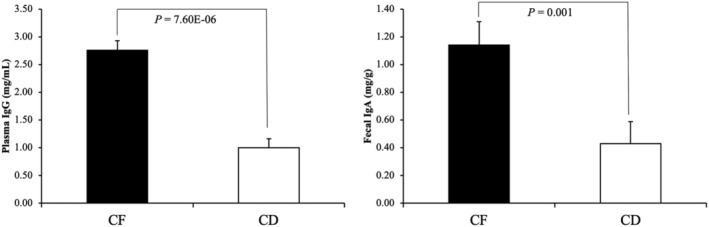
The concentrations of plasma IgG and fecal IgA were in colostrum‐fed (CF) and colostrum‐deprived (CD) piglet groups at 21 days of age. Data are shown as the means ± SEM. Modified from Ogawa, Tsukahara, et al. (2016)

Rooke et al. (2003) concluded that the volume of colostrum ingested during the first 24 h of life could affect the ability of piglets to produce IgG around weaning time. They demonstrated a positive correlation of the concentration of plasma IgG at weaning with the volume of colostrum ingested during the first 24 h of life. By measuring the maternal‐derived, virus‐specific IgG, Rooke et al. showed that there was a lesser contribution of colostrum‐derived IgG to the amount of plasma IgG in piglets at weaning. Based on this evidence, we conducted a study to evaluate the effect of the colostrum ingested during the first 24 h on the development of the immune systems of piglets (Ogawa, Tsukahara, et al., 2016). In our study, 36 piglets from five litters were divided into colostrum‐fed (CF) and colostrum‐deprived (CD) groups. During the first 24 h post delivery, the CF group was raised normally, that is, ingested colostrum from sows, whereas the CD group was given formula milk. Afterwards, all piglets were allowed to normally suckle milk from their respective sows. Next, the concentrations of fecal IgA and plasma IgG were analyzed around weaning time. We observed significantly lower concentrations of fecal IgA and plasma IgG in the CD group than in the CF group (Figure 3). Moreover, around weaning time, the CD group showed a significantly lower number of plasma B cells (CD21^+^ cells) than did CF group. These observations strongly suggested that the colostrum stimulated the development of both the systemic and the mucosal immunity in piglets.

## 8 CONCLUSIONS

Recent improvement of sow reproductivity has resulted in a significant increase in the total number of piglets born, a trend that still continues (Kemp et al., 2018). For example, in Denmark, from 1996 to 2011, the litter size increased from 11.2 to 14.8 (Rutherford et al., 2013). However, an increase in litter size does not result in an increase in total sow milk yield (Devillers et al., 2007). Rutherford et al. (2013) also found that piglet mortality in Denmark increased from 18.2% to 23.5% during the same period (1996–2011). An increase in the litter size seemingly restricts the access of piglets to sufficient colostrum due to the limited number of teats. Thus, there is a need for the discovery or development of a proper colostrum “replacer” for neonates of highly prolific sows, as sufficient colostrum ingestion is of utmost importance to reduce piglet mortality. A proper colostrum replacer is still unavailable to date, mainly because the components and functions of the porcine colostrum are yet to be fully identified. Therefore, to sustain higher survival rates and weaning body weights of piglets, investigation focusing on the yet‐to‐be discovered components and physiological functions of the porcine colostrum should be conducted.

## CONFLICT OF INTEREST

The authors declare there is no conflict of interests.

## References

[asj13618-bib-0001] Alsaweed, M. , Hartmann, P. E. , Geddes, D. T. , & Kakulas, F. (2015). MicroRNAs in breastmilk and the lactating breast: Potential immunoprotectors and developmental regulators for the infant and the mother. International Journal of Environmental Research and Public Health, 12, 13981–14020. 10.3390/ijerph121113981 26529003 PMC4661628

[asj13618-bib-0002] Bandrick, M. , Pieters, M. , Pijoan, C. , & Molitor, T. W. (2008). Passive transfer of maternal mycoplasma hyopneumoniae‐specific cellular immunity to piglets. Clinical and Vaccine Immunology, 15, 540–543. 10.1128/CVI.00466-07 18184823 PMC2268269

[asj13618-bib-0003] Burrin, D. G. , Shulman, R. J. , Reeds, P. J. , Davis, T. A. , & Gravitt, K. R. (1992). Porcine colostrum and milk stimulate visceral organ and skeletal muscle protein synthesis in neonatal piglets. The Journal of Nutrition, 122, 1205–1213. 10.1093/jn/122.6.1205 1375287

[asj13618-bib-0004] Chen, T. , Xi, Q. Y. , Ye, R. S. , Cheng, X. , Qi, Q. E. , Wang, S. B. , Shu, G. , Wang, L. N. , Zhu, X. T. , Jiang, Q. Y. , & Zhang, Y. L. (2014). Exploration of microRNAs in porcine milk exosomes. BMC Genomics, 15, 100. 10.1186/1471-2164-15-100 24499489 PMC4008308

[asj13618-bib-0005] Chen, T. , Xie, M.‐Y. , Sun, J.‐J. , Ye, R.‐S. , Cheng, X. , Sun, R.‐P. , Wei, L. M. , Li, M. , Lin, D. L. , Jiang, Q. Y. , Xi, Q. Y. , & Zhang, Y.‐L. (2016). Porcine milk‐derived exosomes promote proliferation of intestinal epithelial cells. Scientific Reports, 6, 33862. 10.1038/srep33862 27646050 PMC5028765

[asj13618-bib-0006] Clarke, R. M. , & Hardy, R. N. (1971). Histological changes in the small intestine of the young pig and their relation to macromolecular uptake. Journal of Anatomy, 108, 63–77. 5543214 PMC1234227

[asj13618-bib-0007] Csapó, J. , Martin, T. G. , Csapó‐Kiss, Z. S. , & Házas, Z. (1996). Protein, fats, vitamin and mineral concentrations in porcine colostrum and milk from parturition to 60 days. International Dairy Journal, 6, 881–902. 10.1016/0958-6946(95)00072-0

[asj13618-bib-0008] Devillers, N. , Farmer, C. , Le Dividich, J. , & Prunier, A. (2007). Variability of colostrum yield and colostrum intake in pigs. Animal, 1, 1033–1041. 10.1017/S175173110700016X 22444806

[asj13618-bib-0009] Devillers, N. , Le Dividich, J. , & Prunier, A. (2011). Influence of colostrum intake on piglet survival and immunity. Animal, 5, 1605–1612. 10.1017/S175173111100067X 22440352

[asj13618-bib-0010] Donovan, S. M. , McNeil, L. K. , Jimenez‐Flores, R. , & Odle, J. (1994). Insulin‐like growth factors and insulin‐like growth factor binding proteins in porcine serum and milk throughout lactation. Pediatric Research, 36, 159–168. 10.1203/00006450-199408000-00005 7970929

[asj13618-bib-0011] Elliot, J. I. , Senft, B. , Erhardt, G. , & Fraser, D. (1984). Isolation of lactoferrin and its concentration in sows' colostrum and milk during a 21‐day lactation. Journal of Animal Science, 59, 1080–1084. 10.2527/jas1984.5941080x 6511679

[asj13618-bib-0012] Evans, P. A. , Newby, T. J. , Stokes, C. R. , & Bourne, F. J. (1982). A study of cells in the mammary secretions of sows. Veterinary Immunology and Immunopathology, 3, 515–527. 10.1016/0165-2427(82)90017-4 7147696

[asj13618-bib-0013] Gu, Y. , Li, M. , Wang, T. , Liang, Y. , Zhong, Z. , Wang, X. , Zhou, Q. , Chen, L. , Lang, Q. , He, Z. , Chen, X. , Gong, J. , Gao, X. , Li, X. , & Lv, X. (2012). Lactation‐related microRNA expression profiles of porcine breast milk exosomes. PLoS ONE, 7, e43691. 10.1371/journal.pone.0043691 22937080 PMC3427246

[asj13618-bib-0014] Herpin, P. , Louveau, I. , Damon, M. , & Le Dividich, J. (2005). Chapter 14 Environmental and hormonal regulation of energy metabolism in early development of the pig. In D. G. Burrin & H. J. Mersmann (Eds.), Biology of growing animals. Elsevier. 10.1016/S1877-1823(09)70021-9

[asj13618-bib-0015] Hlavova, K. , Stepanova, H. , & Faldyna, M. (2014). The phenotype and activation status of T and NK cells in porcine colostrum suggest these are central/effector memory cells. The Veterinary Journal, 202, 477–482. 10.1016/j.tvjl.2014.09.008 25438731

[asj13618-bib-0016] Hurley, W. L. (2015). Composition of sow colostrum and milk. In C. Farmer (Ed.), The gestating and lactating sow. Wageningen Academic Publishers.

[asj13618-bib-0017] Jaeger, L. A. , Lamar, C. H. , Bottoms, G. D. , & Cline, T. R. (1987). Growth‐stimulating substances in porcine milk. American Journal of Veterinary Research, 48, 1531–1533. 3314608

[asj13618-bib-0018] Kemp, B. , Da Silva, C. L. A. , & Soede, N. M. (2018). Recent advances in pig reproduction: Focus on impact of genetic selection for female fertility. Reproduction in Domestic Animals, 53(Suppl 2), 28–36. 10.1111/rda.13264 30238653

[asj13618-bib-0019] Kim, S. W. , Hurley, W. L. , Hant, I. K. , & Easter, R. A. (2000). Growth of nursing pigs related to the characteristics of nursed mammary glands. Journal of Animal Science, 78, 1313–1318. 10.2527/2000.7851313x 10834588

[asj13618-bib-0020] Klobasa, F. , Werhahn, E. , & Butler, J. E. (1987). Composition of sow milk during lactation. Journal of Animal Science, 64, 1458–1466. 10.2527/jas1987.6451458x 3583950

[asj13618-bib-0021] Le Dividich, J. , Rooke, J. A. , & Herpin, P. (2005). Nutritional and immunological importance of colostrum for the new‐born pig. Journal of Agricultural Science, 143, 469–485. 10.1017/s0021859605005642

[asj13618-bib-0022] Luise, D. , Cardenia, V. , Zappaterra, M. , Motta, V. , Bosi, P. , Rodriguez‐Estrada, M. T. , & Trevisi, P. (2018). Evaluation of breed and parity order effects on the lipid composition of porcine colostrum. Journal of Agricultural and Food Chemistry, 66, 12911–12920. 10.1021/acs.jafc.8b03097 30350981

[asj13618-bib-0023] Martin, R. , Delgado, S. , Maldonado, A. , Jimenez, E. , Olivares, M. , Fernandez, L. , Sobrino, O. J. , & Rodriguez, J. M. (2009). Isolation of lactobacilli from sow milk and evaluation of their probiotic potential. Journal of Dairy Research, 76, 418–425. 10.1017/S0022029909990124 19640313

[asj13618-bib-0024] Muneta, Y. , Mori, Y. , Shimoji, Y. , & Yokomizo, Y. (2000). Porcine interleukin 18: Cloning, characterization of the cDNA and expression with the baculovirus system. Cytokine, 12, 566–572. 10.1006/cyto.1999.0648 10843730

[asj13618-bib-0025] Nguyen, T. V. , Yuan, L. , Azevedo, M. S. , Jeong, K. I. , Gonzalez, A. M. , & Saif, L. J. (2007). Transfer of maternal cytokines to suckling piglets: In vivo and in vitro models with implications for immunomodulation of neonatal immunity. Veterinary Immunology and Immunopathology, 117, 236–248. 10.1016/j.vetimm.2007.02.013 17403542 PMC4094377

[asj13618-bib-0026] Ogawa, S. , Okutani, M. , Tsukahara, T. , Nakanishi, N. , Kato, Y. , Fukuta, K. , Romero‐Pérez, G. A. , Ushida, K. , & Inoue, R. (2016). Comparison of gene expression profiles of T cells in porcine colostrum and peripheral blood. American Journal of Veterinary Research, 77, 961–968. 10.2460/ajvr.77.9.961 27580107

[asj13618-bib-0027] Ogawa, S. , Tsukahara, T. , Imaoka, T. , Nakanishi, N. , Ushida, K. , & Inoue, R. (2016). The effect of colostrum ingestion during the first 24 hours of life on early postnatal development of piglet immune systems. Animal Science Journal, 87, 1511–1515. 10.1111/asj.12573 26990379

[asj13618-bib-0028] Ogawa, S. , Tsukahara, T. , Nishibayashi, R. , Nakatani, M. , Okutani, M. , Nakanishi, N. , Ushida, K. , & Inoue, R. (2014). Shotgun proteomic analysis of porcine colostrum and mature milk. Animal Science Journal, 85, 440–448. 10.1111/asj.12165 24450292

[asj13618-bib-0029] Ogawa, S. , Tsukahara, T. , Tsuruta, T. , Nishibayashi, R. , Okutani, M. , Nakatani, M. , Higashide, K. , Iida, S. , Nakanishi, N. , Ushida, K. , & Inoue, R. (2014). Evaluation of secretion volume and immunoglobulin A and G concentrations in sow colostrum from anterior to posterior teats. Animal Science Journal, 85, 678–682. 10.1111/asj.12211 24798788

[asj13618-bib-0030] Payne, L. C. , & Marsh, C. L. (1962). Absorption of gamma globulin by the small intestine. Federation Proceedings, 21, 909–912. 13942165

[asj13618-bib-0031] Quesnel, H. , Farmer, C. , & Devillers, N. (2012). Colostrum intake: Influence on piglet performance and factors of variation. Livestock Science, 146, 105–114. 10.1016/j.livsci.2012.03.010

[asj13618-bib-0032] Rooke, J. A. , & Bland, I. M. (2002). The acquisition of passive immunity in the new‐born piglet. Livestock Production Science, 78, 13–23. 10.1016/S0301-6226(02)00182-3

[asj13618-bib-0033] Rooke, J. A. , Carranca, C. , Bland, I. M. , Sinclair, A. G. , Ewen, M. , Bland, V. C. , & Edwards, S. A. (2003). Relationships between passive absorption of immunoglobulin G by the piglet and plasma concentrations of immunoglobulin G at weaning. Livestock Production Science, 81, 223–234. 10.1016/S0301-6226(02)00260-9

[asj13618-bib-0034] Rutherford, K. M. D. , Baxter, E. M. , D'Eath, R. B. , Turner, S. P. , Arnott, G. , Roehe, R. , Ask, B. , Sandøe, P. , Moustsen, V. A. , Thorup, F. , Edwards, S. A. , Berg, P. , & Lawrence, A. B. (2013). The welfare implications of large litter size in the domestic pig I: Biological factors. Animal Welfare, 22, 199–218. 10.7120/09627286.22.2.199

[asj13618-bib-0035] Segura, M. , Martínez‐Miró, S. , López, M. J. , Madrid, J. , & Hernández, F. (2020). Effect of parity on reproductive performance and composition of sow colostrum during first 24 h postpartum. Animals, 10(10), 1853. 10.3390/ani10101853 33053679 PMC7601285

[asj13618-bib-0036] Tao, N. , Ochonicky, K. L. , German, J. B. , Donovan, S. M. , & Lebrilla, C. B. (2010). Structural determination and daily variations of porcine milk oligosaccharides. Journal of Agricultural and Food Chemistry, 58, 4653–4659. 10.1021/jf100398u 20369835 PMC2882034

[asj13618-bib-0037] Trevisi, P. , Luise, D. , Won, S. , Salcedo, J. , Bertocchi, M. , Barile, D. , & Bosi, P. (2020). Variations in porcine colostrum oligosaccharide composition between breeds and in association with sow maternal performance. Journal of Animal Science and Biotechnology, 11, 21. 10.1186/s40104-020-0430-x 32190297 PMC7066846

[asj13618-bib-0038] van Niel, G. , D'Angelo, G. , & Raposo, G. (2018). Shedding light on the cell biology of extracellular vesicles. Nature Reviews Molecular Cell Biology, 19, 213–228. 10.1038/nrm.2017.125 29339798

[asj13618-bib-0039] Waidyatillake, N. T. , Stoney, R. , Thien, F. , Lodge, C. J. , Simpson, J. A. , Allen, K. J. , Abramson, M. J. , Erbas, B. , Svanes, C. , Dharmage, S. C. , & Lowe, A. J. (2017). Breast milk polyunsaturated fatty acids: Associations with adolescent allergic disease and lung function. Allergy, 72, 1193–1201. 10.1111/all.13114 28027401

[asj13618-bib-0040] Wang, T. , & Xu, R. J. (1996). Effects of colostrum feeding on intestinal development in newborn pigs. Biology of the Neonate, 70, 339–348. 10.1159/000244385 9001695

[asj13618-bib-0041] Ward, L. A. , Rich, E. D. , & Besser, T. E. (1996). Role of maternally derived circulating antibodies in protection of neonatal swine against porcine group A rotavirus. The Journal of Infectious Diseases, 174, 276–282. 10.1093/infdis/174.2.276 8699055

[asj13618-bib-0042] Watorek, W. (2003). Azurocidin—Inactive serine proteinase homolog acting as a multifunctional inflammatory mediator. Acta Biochimica Polonica, 50, 743–752. 10.18388/abp.2003_3665 14515154

[asj13618-bib-0043] Wei, J. , Wang, Z. A. , Wang, B. , Jahan, M. , Wang, Z. , Wynn, P. C. , & Du, Y. (2018). Characterization of porcine milk oligosaccharides over lactation between primiparous and multiparous female pigs. Scientific Reports, 8, 4688. 10.1038/s41598-018-23025-x 29549280 PMC5856818

[asj13618-bib-0044] Westrom, B. R. , Svendsen, J. , Ohlsson, B. G. , Tagesson, C. , & Karlsson, B. W. (1984). Intestinal transmission of macromolecules (BSA and FITC‐labelled dextrans) in the neonatal pig. Influence of age of piglet and molecular weight of markers. Biology of the Neonate, 46, 20–26. 10.1159/000242028 6204696

[asj13618-bib-0045] Widdowson, E. M. , & Crabb, D. E. (1976). Changes in the organs of pigs in response to feeding for the first 24 h after birth. Neonatology, 28, 261–271. 10.1159/000240827 1276297

[asj13618-bib-0046] Williams, P. P. (1993). Immunomodulating effects of intestinal absorbed maternal colostral leukocytes by neonatal pigs. Canadian Journal of Veterinary Research, 57, 1–8. 8431798 PMC1263580

[asj13618-bib-0047] Wuryastuti, H. , Stowe, H. D. , Bull, R. W. , & Miller, E. R. (1993). Effects of vitamin E and selenium on immune responses of peripheral blood, colostrum, and milk leukocytes of sows. Journal of Animal Science, 71, 2464–2472. 10.2527/1993.7192464x 8407659

[asj13618-bib-0048] Xu, R. J. , Sangild, P. T. , Zhang, Y. Q. , & Zhang, S. H. (2002). Chapter 5 Bioactive compounds in porcine colostrum and milk and their effects on intestinal development in neonatal pigs. In R. Zabielski , P. C. Gregory , B. Weström , & E. Salek (Eds.), Biology of growing animals. Elsevier. 10.1016/S1877-1823(09)70121-3

[asj13618-bib-0049] Zeng, B. , Wang, H. , Luo, J. , Xie, M. , Zhao, Z. , Chen, X. , Wang, D. , Sun, J. , Xi, Q. , Chen, T. , & Zhang, Y. (2021). Porcine milk‐derived small extracellular vesicles promote intestinal immunoglobulin production through pIgR. Animals (Basel), 11(6), 1522. 10.3390/ani11061522 34073819 PMC8225040

[asj13618-bib-0050] Zhang, S. , Chen, F. , Zhang, Y. , Lv, Y. , Heng, J. , Min, T. , Li, L. , & Guan, W. (2018). Recent progress of porcine milk components and mammary gland function. Journal of Animal Science and Biotechnology, 9, 77. 10.1186/s40104-018-0291-8 30377527 PMC6196465

